# Adult Lifetime Diet Quality and Physical Performance in Older Age: Findings From a British Birth Cohort

**DOI:** 10.1093/gerona/glx179

**Published:** 2017-10-13

**Authors:** Sian M Robinson, Leo D Westbury, Rachel Cooper, Diana Kuh, Kate Ward, Holly E Syddall, Avan A Sayer, Cyrus Cooper

**Affiliations:** 1 MRC Lifecourse Epidemiology Unit, University of Southampton, UK; 2 NIHR Southampton Biomedical Research Centre, University of Southampton and Southampton University Hospitals NHS Trust, UK; 3 MRC Unit for Lifelong Health and Ageing, University College London, UK; 4 MRC Elsie Widdowson Laboratory, Cambridge, UK; 5 Institute of Neuroscience, Newcastle University, Newcastle upon Tyne; 6 NIHR Newcastle Biomedical Research Centre, Newcastle University and Newcastle upon Tyne Hospitals NHS Foundation Trust, Newcastle upon Tyne; 7 NIHR Musculoskeletal Biomedical Research Unit, Nuffield Orthopaedic Centre, University of Oxford, UK

**Keywords:** Diet, Life course, Ageing, Physical function

## Abstract

**Background:**

Current evidence that links “healthier” dietary patterns to better measured physical performance is mainly from older populations; little is known about the role of earlier diet. We examined adult diet quality in relation to physical performance at age 60–64 years.

**Methods:**

Diet quality was defined using principal component analysis of dietary data collected at age 36, 43, 53, and 60–64. Throughout adulthood, diets of higher quality were characterized by higher consumption of fruit, vegetables, and wholegrain bread. Diet quality scores calculated at each age indicated compliance with this pattern. Physical performance was assessed using chair rise, timed-up-and-go, and standing balance tests at age 60–64. The analysis sample included 969 men and women.

**Results:**

In gender-adjusted analyses, higher diet quality at each age was associated with better measured physical performance (all *p* < .01 for each test), although some associations were attenuated after adjustment for covariates. Diet quality scores were highly correlated in adulthood (0.44 ≤ *r* ≤ 0.67). However, conditional models showed that higher diet quality at age 60–64 (than expected from scores at younger ages), was associated with faster chair rise speed and with longer standing balance time (adjusted: 0.08 [95% CI: 0.02, 0.15] and 0.07 [0.01, 0.14] *SD* increase in chair rise speed and balance time, respectively, per *SD* increase in conditional diet quality; both *p* < .05).

**Conclusions:**

Higher diet quality across adulthood is associated with better physical performance in older age. Current diet quality may be particularly important for physical performance, suggesting potential for improvements in diet in early older age.

Simple measures of muscle mass and function, such as handgrip strength and walking speed, act as biomarkers of ageing and are important predictors of future health and mortality (1). The variability in these measurements seen across the population (2) is therefore a concern. Although some of the observed inter-individual differences are explained by known determinants, such as gender, age, and body build, much of the variability is unexplained (3). This has focused interest on modifiable behavioral factors, linked to lifestyle, and their influence on physical performance in older age (4). The recent description of age-related changes in physiology and function in mid-life, occurring many years before losses in function are observed (5), highlights the need to understand the effects of these factors acting across the life course; recognizing that muscle mass and strength in later life reflect both the peak achieved in early adulthood and the rate of decline in later years (3,5–7).

An important lifestyle influence is nutrition, and a number of candidate nutrients (protein, vitamin D, antioxidants, and n-3 fatty acids) have been linked to differences in muscle mass, strength, and physical performance in older age (8). But much of the evidence is observational, and collinearity between dietary constituents limits causal inferences. This has led to a growing interest in the role of diet quality in maintaining good physical and cognitive function during ageing (9). “Healthier” patterns, which are linked to higher intakes of a range of protective nutrients, as well as non-nutrients (such as polyphenols), have been shown to be associated with higher muscle mass, strength, and better measured physical performance (8). For example, lower rates of decline in physical performance have been described in individuals whose diets comply with a Mediterranean pattern (10–13). However, most of these studies have been carried out in older populations, and little is known about the contribution of differences in diet quality earlier in adult life, or in childhood. A recent analysis of data from an 18-year follow-up of the Nurses’ Health Study has, therefore, provided valuable new evidence; participants with healthier diets (averaged scores for Alternative Healthy Eating Index-2010, AHEI-2010), had a lower risk of self-reported incident physical impairment (Short Form-36 physical function scale, SF36) during the study period, suggesting that lifetime diet quality may have a protective role in preventing or delaying losses of physical function (14). But to understand the importance of diet quality, and to evaluate potential cumulative effects across the life course, longitudinal dietary data collected at different ages are needed. Using data from a British birth cohort study, in which prospective diet records were collected in childhood and adult life (15), we examined adult diet quality (ADQ) and its links to physical performance at age 60–64 years, using three standardized tests of physical performance (chair rising, timed up-and-go [TUG], and standing balance).

## Subjects and Methods

### Study Sample

The MRC National Survey of Health and Development (NSHD) is a longitudinal study based on a socially stratified sample of 5362 births occurring in one week in March 1946 across England, Wales, and Scotland (15). By the time of follow-up in 2006–2010, 718 participants had died, 594 had withdrawn from the study, 567 had emigrated, and 320 were lost to the study. Of the remaining participants, 2229 (78% of those invited) were assessed: 1690 (76%) at clinic and 539 (24%) at home (15,16). The study was conducted according to the guidelines set out in the Declaration of Helsinki; ethical approval for the data collection was obtained from the Greater Manchester Local Research Ethics Committee and the Scotland A Research Ethics Committee.

### Dietary Assessment

Diet was assessed using prospective 5-day food diaries completed by the participants at ages 36 (1982), 43 (1989), 53 (1999), and 60–64 (2006–2010) (17,18). All food and drink items consumed were recorded using household measures; images and notes at the start of the diaries were provided to guide estimation of portion size. At age 60–64, 880 men and 989 women completed at least 3 days of the food diary. Of these participants, 988 (53%) had completed food diaries (for at least 3 days) at every adult assessment. All foods and drinks consumed at each age were allocated to one of the 45 mutually exclusive food groups on the basis of similarity of type of food and nutrient composition [Supplementary Table 1]. The average consumption of each food group (g/day) was calculated for all participants at each age. A principal component analysis of the daily consumption of the 45 food groups at each age was used to examine dietary patterns (19). The first component described a “healthier” profile of foods at each age in adulthood (details are given in Supplementary methods; principal component analysis coefficients are shown in Supplementary Table 2). Pattern scores, defining individual participant’s compliance with the “healthier” dietary pattern, are referred to as “diet quality scores” throughout the paper; a higher score indicated a diet of higher quality.

### Physical Performance Outcomes

Physical performance was assessed by trained nurses following standard protocols using three tests at age 60–64: chair rises, TUG, and standing balance (21,22). The time taken to perform 10 chair rises (rise from a sitting to a standing position and sit back down again) was recorded and used to derive chair rise speed as the number of repetitions per minute. The TUG test required the participant to rise from a chair, walk 3 m at a normal pace, turn around, return to the chair, and sit down; TUG speed was calculated by dividing 6 (distance in meters) by the time taken in seconds. Standing balance time was measured as the length of time a participant could stand on one leg with their eyes closed, up to a maximum of 30 seconds. The present analysis included 969 participants, for whom complete dietary data were available (at each age) and at least one measure of physical performance at age 60–64.

### Participant Characteristics

Height and weight were measured by nurses at age 60–64. Self-reported smoking status (never/ex/current) at age 60–64 was categorized into ever smokers and never smokers. Leisure time physical activity at age 60–64 was assessed by questionnaire; participants were asked how often in the previous month they had participated in any sports, vigorous leisure activities, or exercises and categorized into the following groups: inactive (no participation); moderately active (participated in relevant activities one to four times per month); and active (participated in relevant activities 5 or more times per month). Occupational social class was categorized using the Registrar General’s classification: I; II; III non-manual; III manual; IV; and V. Self-reports of diabetes and doctor diagnosed angina and myocardial infarction from assessments undertaken up to and including age 60–64 were used to distinguish between participants with and without reports of diabetes and cardiovascular disease (7).

### Statistical Analysis

Participant characteristics were described using summary statistics. Pearson correlation coefficients were used to assess stability of diet quality. In addition to diet quality scores assessed at each age, an overall ADQ score was calculated for every participant. Using the quartiles of the diet quality score defined at age 60–64, points were allocated to the quarter of the distribution each participant’s diet quality score was in (1 = lowest quarter, 2 = second, 3 = third, and 4 = highest quarter) at each age. These points were summed across the four ages in adulthood; scores ranged from 4 (poor diet quality across all ages) to 16 (high diet quality across all ages).

Diet quality scores at each age and physical performance measures were standardized (sex-specific) in models to ensure effect sizes were comparable. The variable for standing balance time was positively skewed and was therefore log-transformed after adding one. Height and weight were highly correlated; to avoid multi-collinearity problems, a sex-specific standardized residual of weight-adjusted-for-height was calculated as a measure of adiposity. Separate linear regression models were used to examine the association between diet quality scores at different ages and ADQ, in relation to each physical performance measure at 60–64 years. We considered a number of covariates, informed by previous analyses and published literature (14,22); models were adjusted for gender, age at follow-up, height, weight-for-height residual, smoking history, leisure time physical activity, presence of diabetes, and cardiovascular disease. As diet quality has been shown to differ between men and women (23), we examined potential gender–diet interactions; in gender-adjusted models, the statistical significance (*p* < .05) of interaction effects between gender and diet quality scores was assessed. We evaluated the impact of adjustment for social class in separate models, and we examined whether associations in the analysis sample were generalizable to the wider NSHD cohort. In sensitivity analyses, diet quality scores were derived using the maximum available sample with dietary data at each age and relationships between diet quality at each age and the physical performance measures were re-examined.

Conditional models were used to examine whether higher diet quality than expected at age 60–64, given diet quality at earlier ages, was associated with differences in the physical performance measures at age 60–64. To implement these, residuals, representing conditional diet quality scores, were obtained by regressing diet quality scores at each age in adulthood on the adult diet quality scores at all previous assessments (24). Each physical performance measure was then regressed on all conditional diet quality scores with adjustment for diet quality at age 36 and other participant characteristics.

## Results

### Participant Characteristics

The characteristics of 969 participants who were included in the analysis sample are presented in Table 1. Mean age at clinic visit was 63 years among men and women. Although mean TUG speed was similar among men and women, men recorded slightly longer standing balance times and had faster chair rise speed than women on average.

**Table 1.** Characteristics of the NSHD Participants Included in the Analysis.

	Men (*N* = 428)	Women (*N* = 541)
Age at clinic visit (y)^†^	63.2 (1.1)	63.3 (1.1)
Height (m)^†^	1.75 (0.07)	1.62 (0.06)
BMI (kg/m^2^)^†^	27.4 (3.9)	27.1 (4.8)
Ever smoked regularly^‡^	285 (69.0%)	318 (60.8%)
Leisure time physical activity participation^‡^		
None	264 (63.0%)	309 (58.2%)
One to four times/mo	55 (13.1%)	92 (17.3%)
Five or more/mo	100 (23.9%)	130 (24.5%)
Occupational class^‡^		
I—Professional	67 (15.7%)	11 (2.0%)
II—Intermediate	185 (43.3%)	204 (37.7%)
III—Skilled (nonmanual)	42 (9.8%)	210 (38.8%)
III—Skilled (manual)	94 (22.0%)	36 (6.7%)
IV—Partly skilled	32 (7.5%)	58 (10.7%)
V—Unskilled	7 (1.6%)	22 (4.1%)
Diabetes^‡^	28 (6.7%)	24 (4.6%)
Angina/myocardial infarction^‡^	28 (7.0%)	15 (3.0%)
TUG speed (m/s)^†^	0.71 (0.15)	0.69 (0.15)
Chair rise speed (stands/min)^†^	26.8 (7.2)	25.7 (8.0)
Standing balance time (s)^§^	3.8 (2.6, 5.5)	3.5 (2.5, 5.4)

Note: ^†^Mean (standard deviation); ^‡^*n* (%); ^§^median (interquartile range); TUG: timed up-and-go.

Compared with the participants who were assessed at the most recent follow-up of the cohort but were not included in the analysis sample due to incomplete dietary or physical performance data, men and women in the analysis sample had lower mean body mass index and longer standing balance times (*p* < .05 for all); chair rise and TUG speeds did not differ significantly between the two samples (*p* > .05). Women in the analysis sample were more likely to have never smoked, to have engaged in physical activity at least once per month, and to be of higher social class (*p* < .05); there were no differences in these characteristics among men.

### Diet Quality

**Figure 1.**
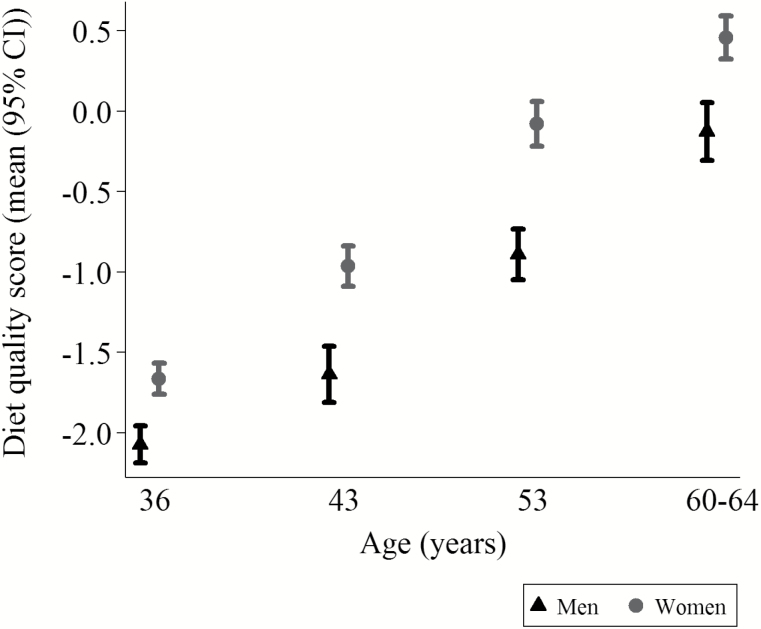
Diet quality scores at ages 36 to 60–64 years.

Mean diet quality score increased from age 36 to 60–64 among men and women (Figure 1). Women had higher diet quality scores than men at all ages in adulthood (*p* < .001). However, although mean diet quality scores increased across adulthood, the correlations between scores at all ages in adulthood were reasonably high (0.44 ≤ *r* ≤ 0.67), indicating stability in ranking of participants in terms of their diet quality between 36 and 60–64 years (Supplementary Table 3). At all ages, increasing diet quality scores were associated with greater consumption of fresh fruit, leafy vegetables, and wholegrain bread, but lower consumption of white bread, added sugar, and processed meat (all *p*-values for trends < .001; Supplementary Table 4). The differences were large such that three- to fourfold differences in consumption were observed for many of these foods across the range of diet quality scores.

### Relationship Between Diet Quality in Adulthood and Physical Performance Measures

The associations between diet quality in adulthood and physical performance at age 60–64 are presented in Table 2. There was little evidence of gender-diet interactions (data not shown); men and women were, therefore, pooled for all analyses. In the gender-adjusted analysis, higher diet quality scores at each age were associated with better measured performance in the three tests (*p* < .01). These associations were mostly robust to adjustment (gender, age at follow-up, height, weight-for-height residual, smoking history, physical activity, diabetes, and cardiovascular disease) (*p* < .05), with the exception of the associations regarding diet quality at age 43, and the association between diet quality at age 53 and TUG speed. There were consistent positive associations between diet quality and physical performance in the cross-sectional associations at 60–64 years, and using the overall ADQ score. The size of effects was meaningful; for example, compared with individuals in the bottom quartile of diet quality at 60–64 years, those in the top quartile performed 2.37 (95% CI: 0.96, 3.79) additional chair rises per minute in gender-adjusted analysis and 1.75 (0.28, 3.23) additional chair rises in fully adjusted analyses (data not shown). Further adjustment for social class attenuated the associations between diet quality at younger ages and physical performance at age 60–64 so that the associations with diet quality at age 53 and between ADQ score and chair rise and TUG speed were no longer statistically significant (data not shown). However, the cross-sectional associations between diet quality at age 60–64 and both chair rise speed and standing balance time were robust to adjustment (both, *p* = .004); TUG was of borderline significance (*p* = .052). Sensitivity analyses, which included all participants who provided dietary data at each age, showed comparable associations between diet quality scores at each age and the physical performance outcomes to the analysis sample (Supplementary Table 5).

**Table 2.** *SD* Difference in Physical Performance Measures at 60–64 years per *SD* increase in Diet Score^†^ at Each Age and Per Unit Increase in Adult Score.

		Chair rise speed		Standing balance time		TUG speed	
Age (y)	M^§^	Estimate (95% CI)	*p*	Estimate (95% CI)	*p*	Estimate (95% CI)	*p*
36	1	0.16 (0.09, 0.22)	<.001	0.12 (0.06, 0.19)	<.001	0.11 (0.04, 0.17)	.002
	2	0.11 (0.05, 0.18)	.001	0.11 (0.05, 0.18)	.001	0.08 (0.01, 0.15)	.029
43	1	0.09 (0.03, 0.16)	.005	0.09 (0.03, 0.16)	.004	0.09 (0.02, 0.15)	.009
	2	0.05 (−0.02, 0.11)	.147	0.06 (0.00, 0.13)	.066	0.05 (−0.02, 0.12)	.185
53	1	0.13 (0.07, 0.20)	<.001	0.11 (0.04, 0.17)	.001	0.11 (0.04, 0.17)	.001
	2	0.08 (0.01, 0.15)	.019	0.08 (0.02, 0.15)	.013	0.06 (−0.01, 0.13)	.092
60–64	1	0.16 (0.10, 0.22)	<.001	0.16 (0.10, 0.22)	<.001	0.12 (0.06, 0.19)	<.001
	2	0.12 (0.06, 0.19)	<.001	0.12 (0.05, 0.18)	<.001	0.09 (0.02, 0.16)	.012
ADQ^‡^	1	0.05 (0.03, 0.07)	<.001	0.05 (0.03, 0.07)	<.001	0.04 (0.02, 0.06)	<.001
	2	0.03 (0.01, 0.05)	.013	0.04 (0.02, 0.07)	<.001	0.03 (0.00, 0.05)	.033

*Note:* ^†^Diet quality scores defined using food consumption data collected at each age and coefficients from the principal component analysis of the dietary data collected at 60–64 years; ^‡^Adult Diet Quality Scores (ADQ), where individuals’ scores were coded from 1 (lowest quartile) to 4 (highest quartile) between age 36 and age 60–64 years and summed to yield a score from 4 to 16 (quartile boundaries based on diet score at 60–64 years); ^§^Model 1: adjusted for gender; Model 2: adjusted for gender, age at follow-up, height, weight-for-height-residual, smoking history, physical activity, diabetes, and cardiovascular disease. *SD*: standard deviations; *p*: *P*-value.

Because there was tracking of diet quality across adulthood, we used conditional models to explore whether higher diet quality than predicted, based on earlier diet quality scores, was associated with better physical performance. Using these models, higher diet quality than expected at age 60–64, when taking into account earlier diet quality, was associated with faster chair rise speed and with longer standing balance time (Table 3). These relationships were robust in the fully adjusted analysis and when additionally adjusted for social class (data not shown).

**Table 3:** *SD* Difference in Physical Performance Measures at Age 60–64 years Per *SD* Increase in Conditional Diet Quality at Age 60–64 years.

Model	Chair rise speed (*SD*s)		Standing balance time (*SD*s)		TUG speed (*SD*s)	
	(95% CI)	*p*-value	(95% CI)	*p*-value	(95% CI)	*p*-value
1	0.08 (0.02, 0.15)	.010	0.10 (0.04, 0.17)	.001	0.06 (0.00, 0.12)	.067
2	0.08 (0.02, 0.15)	.011	0.07 (0.01, 0.14)	.027	0.06 (−0.01, 0.12)	.091

*Note:* Diet quality defined using food consumption data collected at each age and coefficients from the principal component analysis of the dietary data collected at 60–64 years; estimates represent the *SD* change in physical performance measure per sex-specific *SD* increase in diet quality at age 60–64 years, conditional on diet quality at all previous ages; Model 1: adjusted for gender; Model 2: adjusted for gender, age at follow-up, height, weight-for-height residual, smoking history, physical activity, diabetes, and cardiovascular disease.

## Discussion

We have used prospective diet records, collected at four points between the ages of 36 and 60–64, to examine associations between diet quality across adulthood and measured physical performance in older age. Diets of higher quality, characterized by higher consumption of fruit and vegetables and wholegrain bread, and lower consumption of white bread, potato products, added sugar, and processed meat, were positively associated with all measures of physical performance at 60–64 years. This was a consistent finding for diet quality defined at different ages and using an index of overall diet quality in adulthood and was largely robust to adjustment for other participant characteristics, which included physical activity and smoking status. Comparable associations to the analysis sample were observed when dietary data were analyzed for all available participants, and findings were similar in men and women. However, the strongest associations were observed in the cross-sectional analyses of diet quality at 60–64 years, in relation to contemporary measures of physical performance. Furthermore, our conditional analyses showed higher diet quality than expected at age 60–64 (when taking into account earlier diet quality) was associated with faster chair rise speed and with longer standing balance time. This may suggest that changes in food choice, to improve overall diet quality in later life, could have potential to improve physical performance—and to contribute to healthier ageing.

There is some interest in the effects of long-term exposure to dietary patterns across the life course, as influences on health in later life. For example, in a large population of adults studied in the China Health and Nutrition Survey, “healthier” dietary trajectories in mid-life were associated with lower HbA1C at 15-year follow-up, even if contemporary dietary pattern scores were similar (25). This suggests that the cumulative effects of exposure linked to diabetes risk in later life may be more important than current diet. Similarly, using data from the NSHD to examine long-term effects of dietary patterns on bone health in older age, Ward and colleagues found that the trajectory of a protein-calcium–potassium rich dietary pattern between 36 and 60–64 years was associated with higher bone mineral content across all skeletal sites in women (26).

Our finding that higher adult diet quality is related to better measured physical performance in older age is consistent with other evidence, although there are few data from other longitudinal studies, with repeat assessments of dietary intake throughout adulthood, to compare directly with our study. The most comparable prospective cohort is the Whitehall cohort in the UK, in which fruit and vegetable consumption, an indicator of diet quality, was reported 17, 10, and 5 years before an assessment of physical performance (at mean age 65.9 ± 5.9 years) (27). The findings were similar; low fruit and vegetable consumption (less than two portions per day) recorded at each wave, and a longer period of low fruit and vegetable consumption in adult life, was associated with slower walking speed at follow-up. In the Whitehall study, there was evidence of an accumulation of risk for slow walking speed that was associated with low fruit and vegetable consumption. Although we found graded differences in measured physical performance in relation to overall ADQ, which may be consistent with cumulative effects (Table 2), the tracking of diet quality throughout adulthood made this difficult to assess in our study. Furthermore, the observed improvements in measured physical performance, in relation to improvements in diet quality closer to the time of measurement, seen in the conditional analyses, may point more to the importance of current diet. However, further data are needed to confirm this.

The compilation of dietary data over long time periods is affected by background changes in food habits and food availability. Using the longitudinal dietary data collected in this survey, we were able to describe improvements in diet quality between 1982 and the most recent data collection in 2006–2010; although women had higher diet quality scores at each age in adulthood, the changes over time were evident in both genders. These changes are explained by trends in food consumption that have been described previously (17); consumption of less healthy foods (including white bread, added sugar, and processed meat) fell, whereas healthy foods (including vegetables, wholemeal bread, reduced-fat milk) increased. This may reflect participants’ increasing adherence to healthy eating guidance (promoted consistently in the UK since the 1980s), together with changes to the food supply (such as the availability of reduced fat milk) (28). To ensure comparability of patterns over time, we used fixed coefficients from the principal component analysis of the dietary data collected at 60–64 years (20); using this approach, we were able to show stability in ranking of participants in terms of diet quality through adulthood, alongside the background changes in diet that were taking place.

A strength of this study is the availability of prospective dietary data, assessed and analyzed using consistent methods across adulthood. It is also important that we examined objective measures of physical performance. But it is a limitation of the study that a sub-sample of the original cohort, who provided dietary data at each stage of the survey, was included in this analysis, and that they differed in some characteristics from the remainder of the cohort. For example, when compared with other participants who were studied at 60–64 years who did not have complete dietary data, average body mass index was lower among the participants in the analysis sample. Although this may have implications for the external validity of the findings presented, we make internal comparisons in our analyses, and it is unlikely that these differences would explain the data we present. Furthermore, sensitivity analyses, using diet quality scores, derived using the maximum available sample with dietary data at each age, showed similar associations to the analysis sample. A second limitation is that we used self-reported dietary data, for which there are concerns regarding measurement error (29). However, this may be less important for the description of overall dietary patterns; importantly, comparable patterns have been reported using different dietary assessment methods (30,31). Thirdly, although we observed links between lower diet quality at younger ages in adulthood and poorer measured physical performance when the NSHD participants were aged 60–64, we cannot exclude the possibility that reverse causation could explain or contribute to these associations. Finally, although in observational data there is potential for residual confounding, and additionally, we cannot account for measurement error in the covariates considered, there are sound mechanistic reasons to link higher diet quality to better physical performance in older age; this pattern would be expected to provide higher intakes of a range of nutrients (protein, vitamin D, and antioxidants) (23), which, in turn, are linked to positive effects on muscle mass and strength and physical performance in older age (8).

Our findings link quality of diet to better physical performance in older age, suggesting that improvement in diet quality has the potential to yield gains in function. If these observational findings are confirmed in other studies, they have important public health implications: positive changes in dietary patterns in early older age could have benefits for physical performance and healthier ageing.

## Supplementary Material

Supplementary data is available at *The Journals of Gerontology, Series A: Biological Sciences and Medical Sciences* online.

## Funding

The NSHD is funded by the UK Medical Research Council (MRC). The MRC also supports RC and DK (program code MC_UU_12019/4), and KW (U105960371). SMR, LW, HES, AAS, and CC are supported by the MRC (MC_UP_A620_1015, MC_UU_12011/2) and the University of Southampton, UK.

## Author Contributions

The authors’ responsibilities were as follows: DK and RC were responsible for the design and conduct of the NSHD. SMR, AAS, HES, and LW planned the analyses. LW conducted the statistical analyses with support from HES and RC. SMR and LW wrote the first draft of the manuscript. All authors read and approved the final version of the manuscript.

## Supplementary Material

Supplementary Methods
