# Supplementary material for: Adult Lifetime Diet Quality and Physical Performance in Older Age: Findings From a British Birth Cohort
Source: J Gerontol A Biol Sci Med Sci. 2017 Oct 13;73(11):1532–7. doi: 10.1093/gerona/glx179 (PMC6104809; doi:10.1093/gerona/glx179)
Supplement: Supplementary Methods [file glx179_suppl_supplementary_information.docx]

**Supplementary methods**

*Diet quality scores*

A principal component analysis (PCA) of the daily consumption of the 45 food groups at each age was used to examine dietary patterns [19]. The first component described a ‘healthier’ profile of foods at each age in adulthood (Supplementary Table 2); the pattern of foods was characterized by higher consumption of fruit, vegetables and wholegrain bread, and lower consumption of white bread, potato products, added sugar and processed meat. Pattern scores, defining individual participant’s compliance with the ‘healthier’ dietary pattern, can be calculated by multiplying their recorded consumption of each food group by its PCA coefficient (Supplementary table 2), and summed. In this study, to enable comparison of pattern scores determined at different ages on a common scale (thus reflecting the same dietary pattern), the PCA coefficients were ‘fixed’ for the pattern score calculations [20]; PCA coefficients determined at 60-64 years were used to calculate scores at every age. The pattern scores are referred to as ‘diet quality scores’ throughout the paper; a higher score indicated a diet of higher quality.

**Supplementary Table 1: Food groups used in the Principal Component Analysis**

| **Food group** |  |
| --- | --- |
| Brown and wholemeal bread | Brown bread, granary, wheatgerm and wholemeal |
| White bread |  |
| Breakfast cereal | Oat based, low fibre and high fibre breakfast cereal |
| Crispbreads | Crispbreads such as Rivetas, Grissini and Toast Melba |
| Rice and pasta | Rice, pasta and dishes |
| Pizza |  |
| Cakes and biscuits | Biscuits, cereal bars, sweet cereal products (pastries, buns) |
| Puddings | Cereal and milk based puddings, ice cream and dairy desserts |
| Full fat milk |  |
| Reduced fat milk | 1% fat, semi-skimmed, skimmed animal and plant based |
| Cream and fromage frais |  |
| Cheese and cottage cheese |  |
| Yoghurts | Full fat and reduced fat yoghurts and drinking yoghurts |
| Eggs and egg dishes |  |
| Full fat spread | Animal based fats, butter and plant based fats (full fat) |
| Reduced fat spread | Plant based fats (low fat and reduced fat) |
| Frying fats and oils |  |
| Poultry (chicken, turkey) |  |
| Red meat | Beef, lamb, pork and other red meat |
| Processed meat | Bacon, ham, sausages, burgers, meat pies |
| Offal | Liver and other offal dishes such as haggis and faggots |
| White fish and shell fish |  |
| Oily fish |  |
| Vegetables - brassicaceae |  |
| Tomatoes | Tomatoes, including puree, sun dried, raw and canned |
| Leafy vegetables | Yellow, red and dark green leafy vegetables |
| Other vegetables |  |
| Baked beans, pulses and lentils |  |
| Potatoes |  |
| Potato products, includes French fries, | Potato products including chips, wedges and instant mash |
| Savoury snacks | Savoury snacks (cereal, potato and vegetable based) |
| Fresh fruit |  |
| Pure fruit juice and smoothies |  |
| Nuts and seeds | Nuts and seeds (including peanut butter) |
| Dried fruit |  |
| Cooked and tinned fruit |  |
| Sweets and confectionery | Confectionery, chocolate based products, sorbets, lollies |
| Sweet spreads, honey, jam | Jam, marmalade and other sugars such as syrups and honey |
| Added sugar | Pure sugar |
| Carbonated soft drinks |  |
| Non-carbonated (fruit based) drinks | Fruit based drinks, fruit juice drinks, fruit cordials |
| Water | Water (still, tap, sparkling and flavoured) |
| Tea and coffee |  |
| Powdered beverages, cocoa | Powdered beverages such as cocoa, Horlicks |
| Miscellaneous | Including cooking sauces and accompaniments |

**Supplementary Table 2. Coefficients for the first principal component from analysis of participants’ dietary data at each age diet was assessed**

|  | **Age (years)** | | | | |
| --- | --- | --- | --- | --- | --- |
|  |  | **36** | **43** | **53** | **60-64** |
| White bread |  | **-0.36** | **-0.26** | **-0.30** | **-0.24** |
| Brown and wholemeal bread |  | 0.17 | **0.22** | **0.21** | 0.12 |
| Breakfast cereal |  | 0.05 | 0.14 | 0.16 | 0.18 |
| Crispbreads |  | 0.16 | 0.17 | 0.11 | 0.11 |
| Rice and pasta |  | 0.03 | 0.08 | 0.17 | 0.10 |
| Pizza |  | 0.04 | 0.03 | 0.01 | 0.00 |
| Cakes and biscuits |  | -0.09 | 0.01 | -0.06 | -0.05 |
| Puddings |  | -0.06 | 0.04 | -0.02 | -0.02 |
| Full fat milk |  | -0.12 | -0.19 | -0.11 | -0.09 |
| Reduced fat milk |  | 0.17 | 0.19 | 0.05 | 0.13 |
| Cream and fromage frais |  | 0.02 | 0.10 | 0.07 | 0.05 |
| Cheese and cottage cheese |  | 0.10 | 0.10 | 0.06 | 0.12 |
| Yoghurts |  | 0.19 | **0.23** | **0.21** | **0.22** |
| Eggs and egg dishes |  | -0.09 | -0.03 | -0.09 | 0.01 |
| Full fat spread |  | **-0.34** | -0.14 | -0.18 | -0.07 |
| Reduced fat spread |  | 0.14 | 0.10 | 0.01 | -0.07 |
| Frying fats and oils |  | -0.17 | 0.15 | 0.09 | 0.14 |
| Poultry (chicken, turkey) |  | 0.02 | 0.07 | 0.03 | -0.03 |
| Red meat |  | -0.08 | -0.10 | -0.16 | -0.15 |
| Processed meat |  | **-0.26** | **-0.22** | **-0.27** | **-0.20** |
| Offal |  | -0.02 | 0.03 | -0.04 | -0.01 |
| White fish and shell fish |  | -0.09 | -0.07 | 0.04 | 0.02 |
| Oily fish |  | 0.08 | 0.16 | 0.16 | **0.22** |
| Vegetables - brassicaceae |  | 0.03 | 0.15 | 0.06 | 0.12 |
| Tomatoes |  | 0.06 | 0.18 | 0.17 | **0.24** |
| Leafy vegetables |  | 0.10 | **0.26** | **0.23** | **0.27** |
| Other vegetables |  | 0.12 | **0.26** | **0.28** | **0.33** |
| Baked beans, pulses and lentils |  | -0.16 | -0.01 | -0.05 | 0.08 |
| Potatoes |  | **-0.39** | -0.04 | -0.08 | 0.01 |
| Potato products, includes French fries, |  | 0.00 | **-0.26** | **-0.24** | -0.19 |
| Savoury snacks |  | -0.08 | -0.01 | -0.04 | 0.02 |
| Fresh fruit |  | **0.29** | **0.33** | **0.34** | **0.34** |
| Pure fruit juice and smoothies |  | 0.04 | 0.05 | 0.17 | 0.16 |
| Nuts and seeds |  | 0.03 | 0.11 | 0.09 | **0.20** |
| Dried fruit |  | 0.10 | 0.13 | 0.15 | **0.21** |
| Cooked and tinned fruit |  | -0.02 | 0.12 | 0.11 | 0.16 |
| Sweets and confectionery |  | -0.04 | 0.00 | -0.06 | 0.05 |
| Sweet spreads, honey, jam |  | 0.02 | 0.12 | 0.07 | 0.03 |
| Added sugar |  | **-0.30** | **-0.25** | **-0.23** | -0.17 |
| Carbonated soft drinks |  | -0.02 | -0.01 | -0.02 | -0.10 |
| Non-carbonated (fruit based) drinks |  | 0.13 | 0.09 | -0.03 | 0.01 |
| Water |  | 0.07 | 0.14 | **0.24** | **0.24** |
| Tea and coffee |  | -0.17 | -0.05 | -0.09 | 0.04 |
| Powdered beverages, cocoa |  | 0.01 | 0.04 | 0.05 | 0.05 |
| Miscellaneous |  | -0.04 | 0.09 | 0.07 | 0.05 |
|  |  |  |  |  |  |
| *Variance explained (%)* |  | 7% | 7% | 7% | 7% |
| Bold text used to highlight coefficients with greatest magnitude (≥0.2 or ≤-0.2)  Coefficients were derived using the maximum available sample with dietary data at each age | | | | | |

**Supplementary Table 3. Pearson correlation coefficients for diet quality scores at each age diet was assessed**

|  |  | **Age (years)** |  |
| --- | --- | --- | --- |
|  | **36** | **43** | **53** |
| **Men** |  |  |  |
| **43** | 0.46^1,2^ |  |  |
| **53** | 0.48 | 0.56 |  |
| **60-64** | 0.47 | 0.54 | 0.67 |
|  |  |  |  |

**Women**

| **43** | 0.55 |  |  |
| --- | --- | --- | --- |
| **53** | 0.50 | 0.65 |  |
| **60-64** | 0.44 | 0.58 | 0.66 |

^1^Diet quality scores defined using food consumption data collected at each age and coefficients from the principal component analysis of the dietary data collected at 60-64 years; ^2^Pearson correlation coefficients and all such values. P<0.001 for all associations.

**Supplementary Table 4: Participants’ consumption of selected foods (g/day) according to quarter of the distribution of diet quality score at each age studied**

|  | **Diet quality quarter^1,2^** | **Age (years)** | | | |
| --- | --- | --- | --- | --- | --- |
|  |  | **36** | **43** | **53** | **60-64** |
| **Fresh fruit** | Lowest | 38 (7, 78)^3^ | 41 (14, 82) | 60 (20, 115) | 57 (18, 104) |
|  | Second | 90 (47, 139) | 94 (51, 166) | 137 (84, 213) | 115 (67, 164) |
|  | Third | 183 (117, 236) | 147 (89, 204) | 187 (120, 271) | 143 (90, 212) |
|  | Highest | 201 (72, 305) | 229 (170, 359) | 272 (200, 367) | 219 (153, 292) |
|  |  |  |  |  |  |
| **Leafy vegetables** | Lowest | 10 (3, 19) | 12 (5, 22) | 0 (0, 0) | 0 (0, 0) |
|  | Second | 18 (8, 32) | 20 (12, 34) | 0 (0, 4) | 0 (0, 8) |
|  | Third | 25 (12, 40) | 29 (16, 43) | 1 (0, 11) | 5 (0, 16) |
|  | Highest | 44 (20, 76) | 43 (27, 59) | 8 (0, 28) | 16 (0, 30) |
|  |  |  |  |  |  |
| **Brown and wholemeal bread** | Lowest | 0 (0, 44) | 14 (0, 51) | 0 (0, 29) | 14 (0, 46) |
|  | Second | 53 (14, 105) | 53 (25, 91) | 32 (8, 65) | 29 (4, 55) |
|  | Third | 55 (28, 84) | 62 (29, 93) | 43 (9, 79) | 32 (4, 62) |
|  | Highest | 26 (8, 83) | 54 (28, 86) | 43 (14, 78) | 43 (14, 70) |
|  |  |  |  |  |  |
| **White bread** | Lowest | 78 (42, 118) | 77 (42, 114) | 87 (54, 119) | 67 (30, 99) |
|  | Second | 24 (5, 56) | 35 (12, 68) | 55 (26, 81) | 42 (14, 68) |
|  | Third | 21 (0, 46) | 22 (0, 44) | 39 (14, 71) | 30 (12, 58) |
|  | Highest | 21 (0, 37) | 7 (0, 26) | 22 (7, 48) | 18 (0, 38) |
|  |  |  |  |  |  |
| **Added sugar** | Lowest | 11 (1, 35) | 5 (0, 26) | 5 (0, 23) | 3 (0, 18) |
|  | Second | 3 (0, 11) | 0 (0, 3) | 0 (0, 5) | 1 (0, 8) |
|  | Third | 1 (0, 3) | 0 (0, 2) | 0 (0, 3) | 1 (0, 6) |
|  | Highest | 0 (0, 2) | 0 (0, 2) | 0 (0, 3) | 1 (0, 5) |
|  |  |  |  |  |  |
| **Processed meat** | Lowest | 49 (26, 73) | 52 (27, 87) | 40 (17, 66) | 43 (23, 68) |
|  | Second | 29 (13, 50) | 26 (11, 52) | 29 (11, 49) | 25 (10, 48) |
|  | Third | 20 (8, 35) | 18 (0, 36) | 18 (5, 34) | 21 (9, 39) |
|  | Highest | 17 (0, 64) | 11 (0, 31) | 10 (0, 22) | 12 (0, 27) |
|  |  |  |  |  |  |
| *Number of participants in each quarter of distribution* | *Lowest* | *775* | *591* | *401* | *247* |
|  | *Second* | *159* | *213* | *235* | *247* |
|  | *Third* | *43* | *124* | *183* | *247* |
|  | *Highest* | *11* | *60* | *169* | *247* |

^1^Diet quality defined using food consumption data collected at each age and coefficients from a principal component analysis (PCA) of the dietary data collected at 60-64 years; ^2^Diet quality groups defined using quartiles of the diet score at age 60-64; ^3^Median (interquartile range) and all such values; P-values for trend in consumption across diet quality groups obtained from linear regression models: all foods shown P<0.001.

**Supplementary Table 5: SD difference in physical performance measures at 60-64 years per SD increase in diet score^1^ at each age when based on the maximum sample size (all participants with dietary data at each age:** **1574 participants at age 36; 1562 participants at age 43; 1375 participants at age 53; 1,829 participants at age 60-64)**

|  |  | **Chair rise speed** | | **Standing balance time** | | **TUG speed** | |
| --- | --- | --- | --- | --- | --- | --- | --- |
| **Age (yrs)** | **M^2^** | **Estimate (95%CI)** | **P** | **Estimate (95%CI)** | **P** | **Estimate (95%CI)** | **P** |
| *36* | ***1*** | 0.13 (0.08,0.18) | <0.001 | 0.10 (0.05,0.15) | <0.001 | 0.07 (0.02,0.13) | 0.006 |
|  | ***2*** | 0.10 (0.04,0.15) | 0.001 | 0.08 (0.03,0.14) | 0.004 | 0.06 (0.01,0.12) | 0.026 |
| *43* | ***1*** | 0.08 (0.03,0.13) | 0.001 | 0.08 (0.03,0.13) | 0.001 | 0.08 (0.03,0.13) | 0.001 |
|  | ***2*** | 0.05 (-0.00,0.10) | 0.057 | 0.05 (-0.00,0.10) | 0.071 | 0.04 (-0.01,0.10) | 0.140 |
| *53* | ***1*** | 0.14 (0.08,0.19) | <0.001 | 0.10 (0.05,0.16) | <0.001 | 0.09 (0.03,0.14) | 0.001 |
|  | ***2*** | 0.08 (0.02,0.14) | 0.007 | 0.08 (0.02,0.14) | 0.011 | 0.04 (-0.02,0.09) | 0.169 |
| *60-64* | ***1*** | 0.14 (0.09,0.19) | <0.001 | 0.13 (0.09,0.18) | <0.001 | 0.12 (0.07,0.17) | <0.001 |
|  | ***2*** | 0.10 (0.05,0.15) | <0.001 | 0.10 (0.05,0.15) | <0.001 | 0.07 (0.03,0.12) | 0.003 |

^1^Diet quality scores defined using food consumption data collected at each age and coefficients from the PCA of the dietary data collected at 60-64 years; ^2^ Model 1: adjusted for gender; Model 2: adjusted for gender, age at follow-up, height, weight-for-height-residual, smoking history, physical activity, diabetes and cardiovascular disease. SD: standard deviations; P: P-value
